# Thiazole Antibiotic Thiostrepton Synergize with Bortezomib to Induce Apoptosis in Cancer Cells

**DOI:** 10.1371/journal.pone.0017110

**Published:** 2011-02-18

**Authors:** Bulbul Pandit, Andrei L. Gartel

**Affiliations:** 1 Department of Medicine, University of Illinois at Chicago, Chicago, Illinois, United States of America; 2 Department of Biochemistry and Molecular Genetics, University of Illinois at Chicago, Chicago, Illinois, United States of America; 3 Department of Microbiology and Immunology, University of Illinois at Chicago, Chicago, Illinois, United States of America; Bauer Research Foundation, United States of America

## Abstract

Thiazole antibiotic, thiostrepton was recently identified as proteasome inhibitor. We investigated the therapeutic potential of the combination of thiostrepton and proteasome inhibitor bortezomib (Velcade) on various human tumor cell lines. Combination of sub-lethal concentrations of thiostrepton and bortezomib induced potent apoptosis and inhibition of long-term colony formation in a wide variety of human cancer cell lines. The synergistic relationship between thiostrepton and bortezomib combination was also quantitatively demonstrated by calculating their combination index values that were much lower than 1 in all studied cell lines. The synergy between these drugs was based on their proteasome inhibitory activities, because thiostrepton modification, thiostrepton methyl ester, which did not have intact quinaldic acid ring and did not inhibit proteasome activity failed to demonstrate any synergy in combination with bortezomib.

## Introduction

Combination of targeted drugs/agents has received a consensus as a viable potential strategy for the treatment of malignancy. Treatment with a conjunction of agents that target key cellular mechanism or proteins critical for various cancers are being investigated against a variety of tumors. Combination of drugs allows for their use at subtoxic concentrations and decreases the likelihood of development of resistant cancer cells.

The proteasome is a protein complex that target ubiquitin-tagged proteins for degradation in an ATP-dependent manner. Recent advances in the understanding of the mechanisms of proteasome activity led to the development of proteasome inhibitors as effective drugs against human cancer [1]. Since certain types of cancer rely on a functional proteasome for growth, inhibition of proteasome activity would selectively kill these tumors. Bortezomib (Velcade) is one of the first in class proteasome inhibitors that inhibits the 26S proteasome by binding to the N terminal threonine residues in the active site of the proteasome catalytic region. It is approved for clinical use in cases of relapsed multiple myeloma, but has demonstrated little to no activity for treatment of solid tumors as a single agent. However, by inhibiting the proteasome pathway and subsequent effect of important pathways, bortezomib demonstrates synergistic relationship when combined with other anti-cancer drugs and enhances the efficacy of drugs against malignancy.

In our previous studies we demonstrated that thiazole antibiotics Siomycin A and thiostrepton induce apoptosis in human cancer cells [2], [3] and act as proteasome inhibitors [4]. It has been demonstrated before that combination of two proteasome inhibitors lactacystin and MG132 synergized against prostate cancer cells in vitro [5]. Additionally, synergy was demonstrated by combining bortezomib with curcumin (which demonstrates proteasomal inhibitory activity in addition to other effects) against multiple myeloma cells [6]. Similarly, we have demonstrated that combination of thiostrepton and bortezomib demonstrated strong synergy against prostate cancer [7]. In this study we confirmed that co-treatment of various tumor cell lines of different origin with sub-lethal concentrations of proteasome inhibitors thiostrepton and bortezomib reveals a strong synergy as demonstrated by induction of apoptosis, combination index values and long-term clonogenic assay.

## Results and Discussion

**Figure 1:**
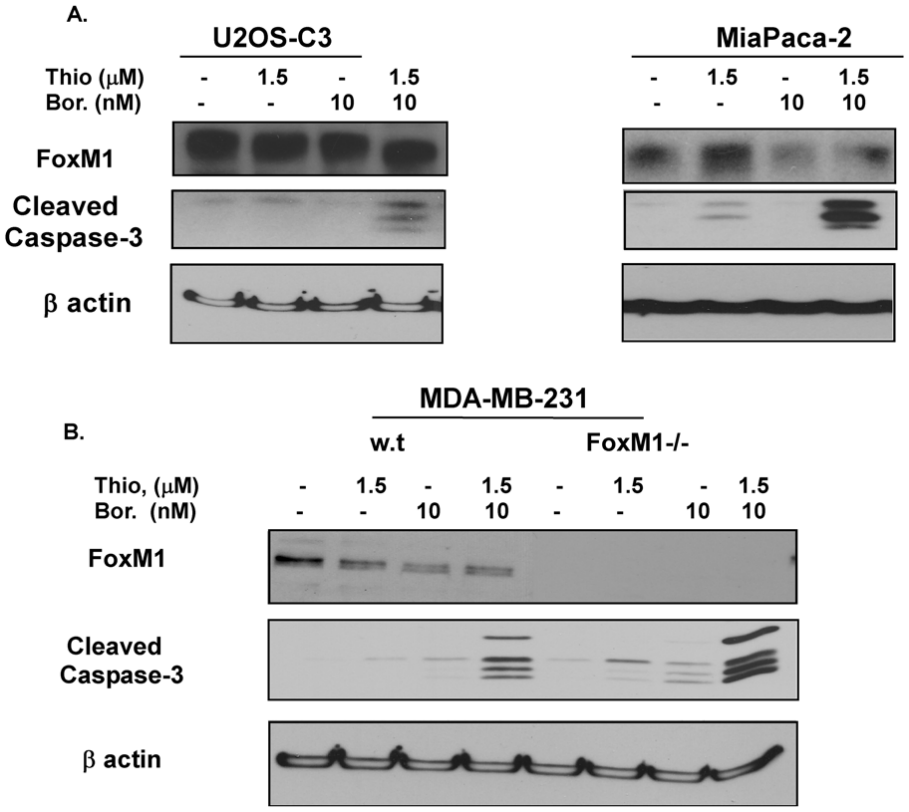
Combination treatment of thiostrepton and bortezomib is synergistic in inducing apoptosis in tumor cells. **A.** Osteosarcoma, U2OS-C3, **B.** MiaPaca-2 pancreatic, **C**. PA-1 ovarian, D. HCT-116 colon, E. MDA-MB-231 breast cancer cells were treated with sub-apoptotic concentrations of thiostrepton, bortezomib or both as shown for 24 hrs, total cell lysates were extracted and immunoblotted with antibodies for cleaved caspase-3 and β-actin.

We showed earlier that proteasome inhibitor thiostrepton inhibited the growth of various cancer cell lines with IC_50_ values of (1–5 µM/L) and induced apoptosis [2], [3]. Bortezomib has been demonstrated to inhibit the viability of tumor cell lines with IC_50_ value of 10–100 nM/L. To determine whether thiostrepton may synergize with bortezomib against human cancer cell lines of different origin we treated multiple human cancer cell lines with either sub-apoptotic concentrations of thiostrepton or bortezomib alone or with combinations of the two for 24 hours and used caspase-3 to serve as an indicator of apoptotic cell death (Figure 1). While treatment with thiostrepton or bortezomib alone induced little or no caspase-3 cleavage in these cells, treatment with combination of these drugs showed potent caspase-3 cleavage in U2OS-C3 osteosarcoma, MiaPaca-2 pancreatic, PA-1 ovarian, HCT116 colon and MDA-MB-231 breast cancer cells (Figure 1), and levels of apoptosis inversely correlated with FoxM1 expression (Figure S1). Since we established earlier synergy between bortezomib and thiostrepton in prostate cancer cells [7], our current data suggest that this effect may have general importance for cancer treatment.

**Figure 2:**
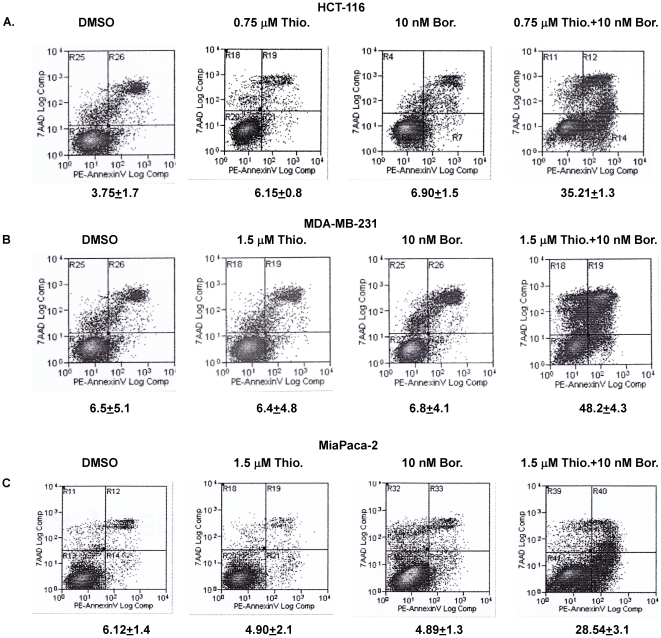
Combination treatment of thiostrepton and bortezomib is synergistic in inducing cell death in tumor cells. **A.** HCT-116, colon, **B.** MDA-MB-231, breast and **C**. MiaPaca-2, pancreatic cancer cells treated with sub-apoptotic concentrations of thiostrepton, bortezomib and thiostrepton/bortezomib combination for 24 hrs, stained with AnnexinV-PE and 7-AAD and analyzed by flow cytometry.

To further demonstrate that combination treatment of thiostrepton and bortezomib induces synergistic apoptosis, we stained these cells (DMSO treated, thiostrepton treated, bortezomib treated and treated together with the two drugs) with annexin V-PE/7AAD and analyzed them by flow cytometry. As shown in Figure 2A, treatment of HCT-116 cells with 0.75 µM thiostrepton or 10 nM bortezomib induced apoptosis of only 6.1% and 6.9% over the control, while treatment with both drugs at the same doses caused 35.2% of cells to undergo apoptosis. Similar synergistic effect of thiostrepton/bortezomib combination was observed by annexin-VPE-7AAD staining in MDA-MB231 breast and MiaPaca-2 pancreatic cancer cells (Figure 2B and C).

**Figure 3:**
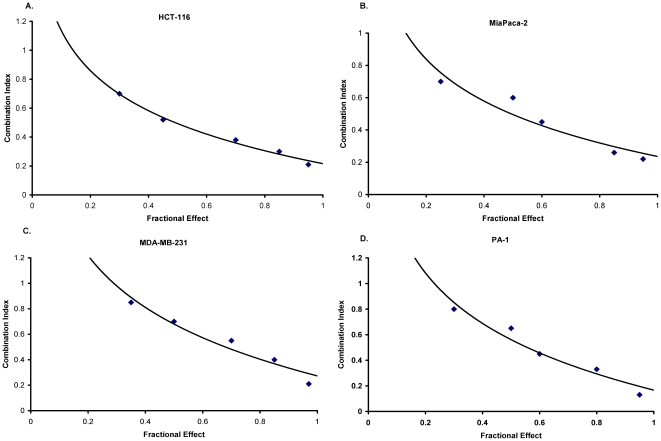
Combination of thiostrepton and bortezomib is synergistic in inhibiting proliferation in cancer cells. Combination index chart for the thiostrepton/bortezomib drug combination was plotted with CI on y axis and fractional effect (FI) on x-axis. Percentage inhibition of proliferation was determined following treatment with a single agent or the thiostrepton/bortezomib combination. Combination index (CI) values of the two agents plotted for HCT-116 (**A**), Miapaca-2 (**B**), MDA-MB231 (**C**) and PA-1 ovarian cells (**D**).

**Figure 4:**
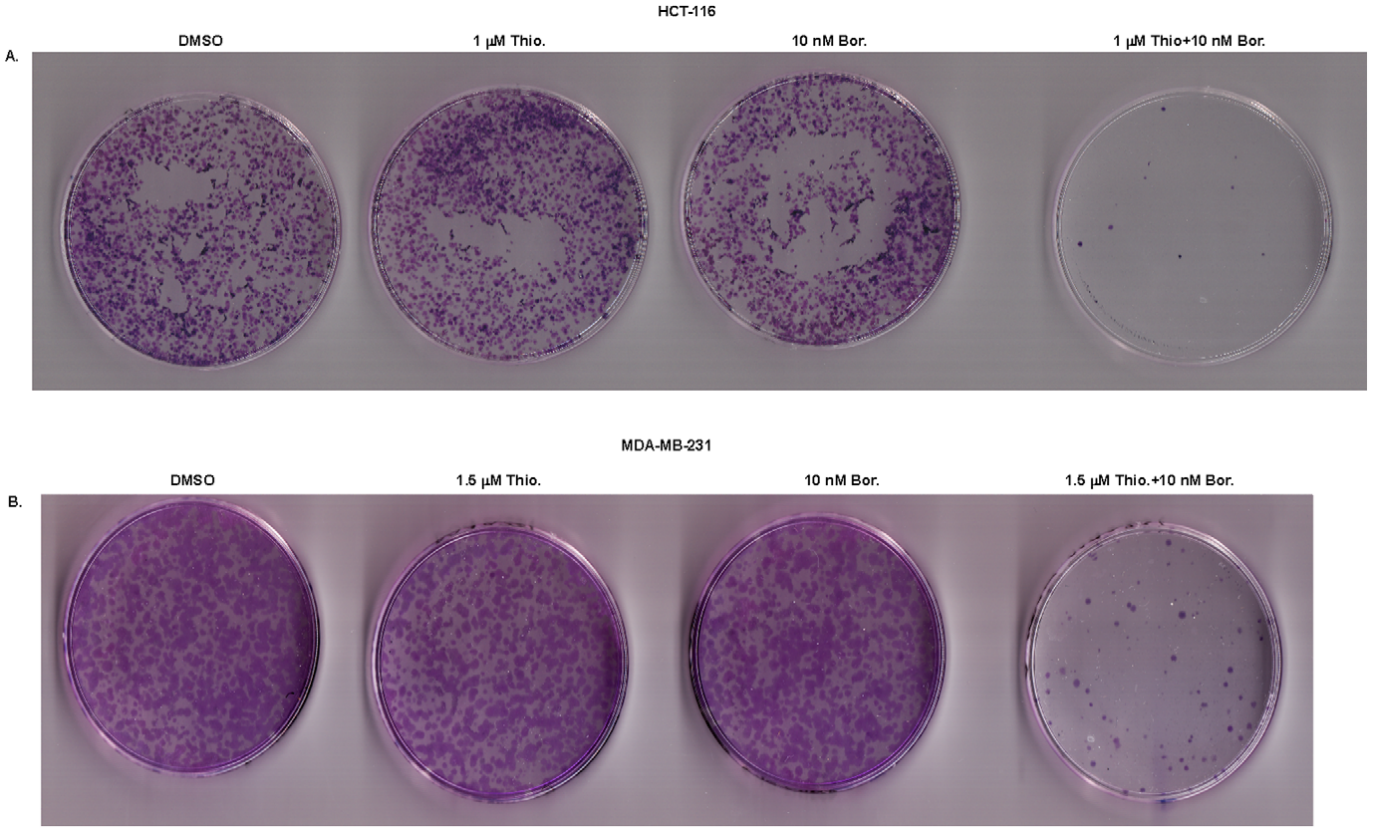
Clonogenic assay shows the long-term effects of combination treatment of human cancer cells with thiostrepton and bortezomib. **A.** Clonogenic assay of HCT-116 cells treated with DMSO or thiostrepton/bortezomib combination for 24 hrs as detailed; a photograph of petri-dishes in a representative experiment is shown. **B.** Clonogenic assay of MDA-MB231 cells treated with DMSO or thiostrepton/bortezomib combinations for 24 hrs.

To quantitatively validate the synergistic nature of the interaction between thiostrepton and bortezomib, we examined cell viability after single and combination drug treatments by using the Chou-Talalay median-effect equation method [8]. CI values below 1 indicate a synergistic anti-proliferative effect, and the CI range values for the combined treatments with thiostrepton/bortezomib in HCT-116, Mia-Paca-2, MDA-MB231 and PA-1 human cancer cell lines were 0.1 to 0.8 (Figure 3A, B, C, D) for fractional effect corresponding to 0.3 to 0.9, suggesting a strong synergistic effect. The long-term effects of combination treatment with thiostrepton and bortezomib were assessed by clonogenic assay. We found that colony formation in HCT-116 colon and MDA-MB 231 breast cancer cells treated with combination of these drugs for 24 hours was suppressed 5- to 10-fold (Figure 4A and B).

**Figure 5:**
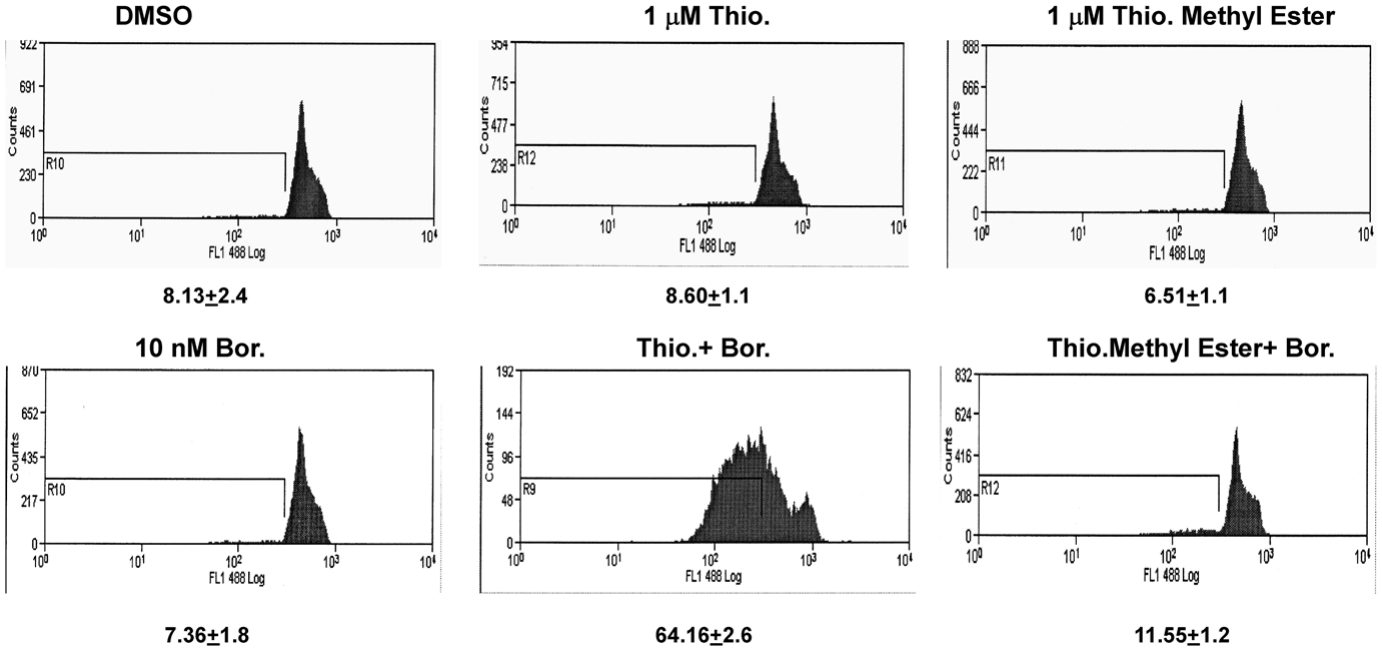
Thiostrepton with intact B ring potentiates chromatin condensation in vitro detection kit. Treatment with combination of thiostrepton/bortezomib results in appearance of sub-G_0_ peak which reduces the fluorescence intensity in HCT-116 cells as opposed to untreated control cells due to fragmentation of chromatin. The shift of the FI was comparable to the positive control provided in the kit (Staurosporine, data not shown). Thiostrepton-methyl ester with open B ring does not affect the fluorescence intensity as opposed to untreated (control) cells.

Following the demonstration of synergy between thiostrepton and bortezomib combination, the significance of proteasomal inhibitory activity of thiostrepton utilized in the combination was investigated. The activity of the combination of thiostrepton methyl ester (open ring inactive structural analog of thiostrepton) and bortezomib combination was compared to thiostrepton and bortezomib. It was demonstrated previously that an intact quinaldic acid macrocycle ring in thiostrepton is required for proteasome inhibitory activity of the thiazole antibiotics [9]. Absence of this ring or presence of an open ring rendered the molecule inactive [10]. In contrast to combination of thiostrepton/bortezomib, bortezomib and thiostrepton methyl ester failed to demonstrate induction of apoptosis, confirming the significance of proteasome inhibitory activity of thiostrepton utilized in the drug combination (Figure 5). Some studies have demonstrated a synergy following co-treatment with proteasome inhibitors, lactacystin and MG132 [5], botezomib and curcumin [6] against cancer. In this study using different methods we report that thiostrepton synergizes with bortezomib following co-treatment with sub-lethal concentrations of the two agents, suggesting that the combination of two proteasome inhibitors to be of potential value as a general strategy against cancer.

## Materials and Methods

### Cell lines and reagents

U2OS-C3 osteosarcoma, HCT116 colon, PA1 ovarian and MiaPaca-2 pancreatic cancer cells were cultured in DMEM medium (Invitrogen). MDA-MB231 cells were grown in RPMI-1640 medium (Invitrogen). The media were supplemented with 10% fetal bovine serum (Atlanta Biologicals) and 1% penicillin-streptomycin (GIBCO) and the cells were kept at 37°C in 5% CO_2_. Cell lines were tested for mycoplasma contamination using MycoAlert Mycoplasma detection kit (Lonza Rockland) and were found to be negative. Thiostrepton was purchased from Sigma, bortezomib was kindly provided by Millennium pharmaceuticals/Takeda. Open ring analog of thiostrepton, thiostrepton methyl ester was kindly provided by Drs. Walsh and Bowers (Harvard University).

### Combination index assay

MTT assay was performed to measure the viability of cells following treatment with single agents or combination of thiostrepton and bortezomib. Each experiment involved treatment with various concentrations of thiostrepton, bortezomib, combination of thiostrepton and bortezomib and a no drug treatment. Additionally, the highest concentrations of thiostrepton, bortezomib and the drug combination were tested in the absence of cells and did not demonstrate any interference with the MTT assay reagents.

3-(4, 5-Dimethylthiazol-2-yl)-2, 5-diphenyltetrazolium bromide (MTT) was procured from Sigma. Cells were plated at a density of 1×10^4^ per well in 200 µL of complete culture medium and treated with thiostrepton alone, bortezomib alone and combination of thiostrepton and bortezomib in 96-well micro titer plates. After incubation for 72 hours at 37°C in a humidified incubator, 10 µL MTT (5 mg/mL in PBS) was added to each well, following which the plate was centrifuged briefly. After careful removal of the medium, 0.1 mL buffered DMSO was added to each well. The absorbance was recorded on a micro-plate reader at the wavelength of 540 nm. In our experiments, the IC_30_, IC_50_, IC_70_, IC_80_, and IC_90_ values (i.e., the drug concentration needed to cause 30%, 50%, 70%, 80%, and 90% reductions in cell viability) were chosen for comparison.

To evaluate the effect of combination treatment with thiostrepton and bortezomib, the combination index (CI) isobologram method of Chou and Talalay was used [8]. This method involves plotting dose–effect curves for each agent and combinations in multiple diluted concentrations by using the median–effect equation and the combination index equation. Combination index values of 1, <1, and >1 indicate an additive effect, synergism, and antagonism, respectively. The combination index values were determined at different effect levels, and the isobolograms plotted.

### Detection of apoptosis

The AnnexinV-PE staining kit (Roche Diagnostic Corp.) was used for the detection of apoptotic bodies following the vendor's protocol. This kit uses a dual-staining protocol in which the cells show fluorescence of Annexin V (apoptotic cells) and fluorescence of 7AAD (necrotic cells or late apoptotic cells). Briefly the tumor cells were grown at a density of 50% confluence in 100-mm culture dishes and were treated with varying concentrations of the drugs for 24 hours. The cells were trypsinized, washed with PBS, and were processed for labeling with annexinV-7AAD. The labeled cells were analyzed by flow cytometry.

### Immunoblotting

Actively dividing cells were seeded into a 100 mm plate at a density of 7.5×10^5^ cells. Cells were treated with bortezomib alone, thiostrepton alone and combination of thiostrepton and bortezomib for 24 hours following which the cells were lysed. Cells were lysed in IP buffer (20 mM HEPES, 1% Triton X-100, 150 mM NaCl, 1 mM EDTA, 1 mM EGTA, 100 mM NaF, 10 mM Na_4_P_2_O_7_, 1 mM sodium orthovanadate, 0.2 mM PMSF supplemented with protease inhibitor tablet (Roche Applied Sciences) and the protein concentration was determined using the Bio-Rad protein assay reagent. Fifty micrograms of the cell lysates were separated by electrophoresis on SDS-polyacrylamide mini gel and transferred to PVDF membrane. Immunoblotting was performed with specific antibodies for cleaved caspase-3 (9664 cell signaling), FoxM1 (kind gift from Dr. Costa's lab) and β-actin (A5441, Sigma).

### Nuclear-ID Green Chromatin Condensation detection

Cells were stained using *in vitro* apoptosis detection kit (Cat# ENZ-51021-K200 Enzo Life Sciences), according to the manufacturer's recommendations. Briefly 3–4×10^5^ cells were plated in 60 mm culture dishes and allowed to grow overnight. Cells were treated with the sub-lethal concentrations of thiostrepton, bortezomib or thiostrepton methyl ester or combination of thiostrepton and bortezomib and thiostrepton-methyl ester and bortezomib. Following overnight incubation of drugs cells were trypsinized and submitted for flow cytometry analysis. Analysis was done by using FL-1 channel of flow cytometer with excitation wavelength of 488 nM. Staurosporine, provided in the kit was used as a positive control.

### Clonogenic survival assay

HCT-116 and MDA-MB231 cells were plated to medium plates at 3×10^5^ cells confluence and treated with combination of thiostrepton and bortezomib for 24 hrs. The cells were then trypsinized, re-suspended in the media and counted. The cells were re-seeded (750 cells per medium plate) and incubated for 10 days. Fresh media was added on the fifth day. On the tenth day, media was removed from the dishes and washed once with ice-cold PBS. The colonies were stained with 2 mls each of 0.25% 1, 9-dimethyl-methylene blue in 50% ethanol for 45 minutes on a rocking platform. The dishes were rinsed three times with PBS and air-dried, and the colonies were counted.

### Statistical analysis

Statistical analysis was performed with Microsoft Excel using the Student *t* test. *P* values of <0.05 were considered to be statistically significant.

## Supporting Information

Figure S1 **Relative levels of FoxM1 may affect the sensitivity to thiostrepton/bortezomib combination treatment in human cancer cells.** U2OS-C3 osteosarcoma and MIA PaCa-2 pancreatic cancer cells were treated with DMSO or indicated concentrations of thiostrepton and borteozomib together for 24 hours. Cell lysates were immunoblotted for FoxM1, cleaved caspase-3 and β-actin as the loading control.
(TIF)
