# Supplementary figures and images for: Thiazole Antibiotic Thiostrepton Synergize with Bortezomib to Induce Apoptosis in Cancer Cells
Source: PLoS One. 2011 Feb 18;6(2):e17110. doi: 10.1371/journal.pone.0017110 (PMC3041825; doi:10.1371/journal.pone.0017110)

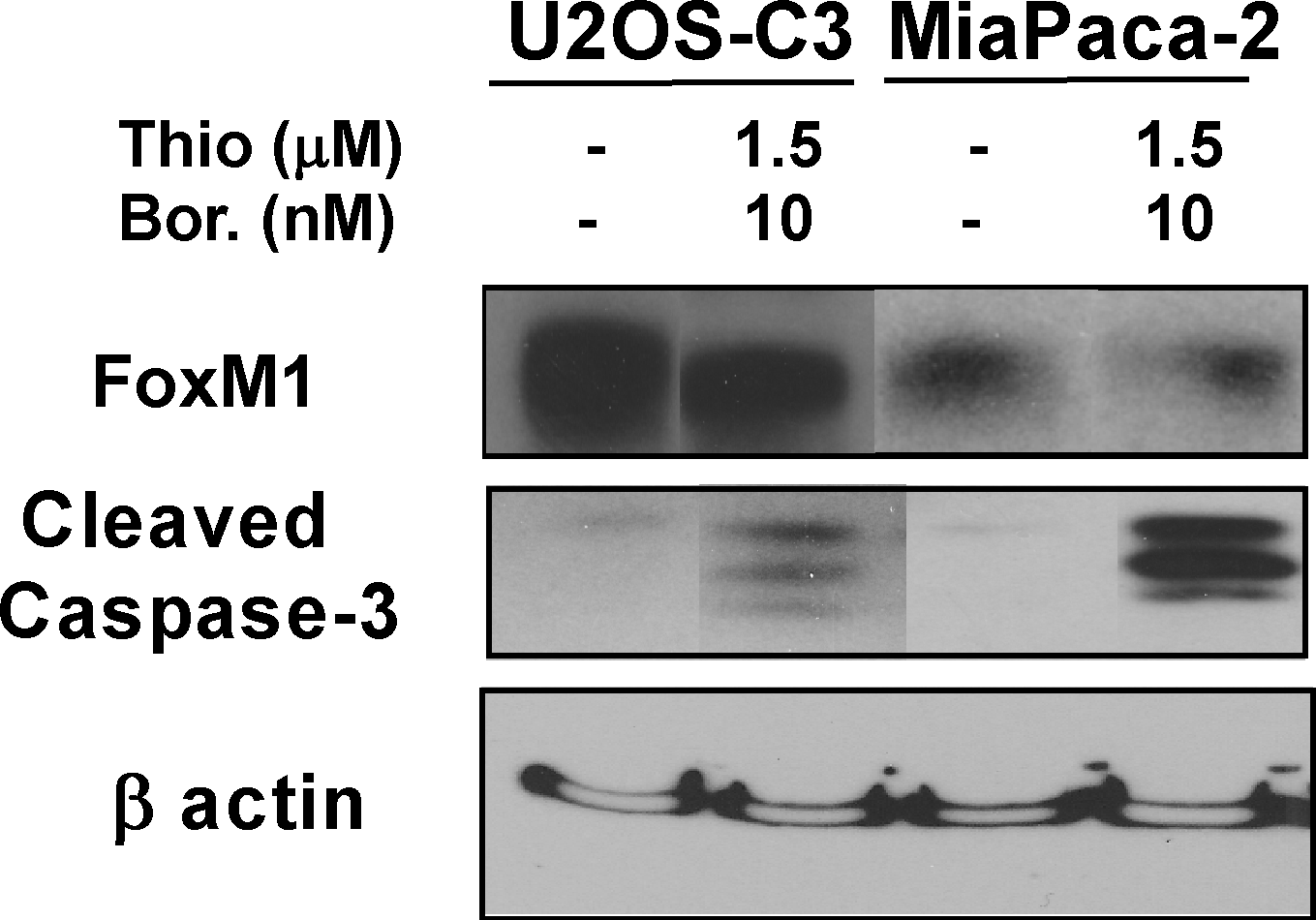

Supplement: Figure S1 — Relative levels of FoxM1 may affect the sensitivity to thiostrepton/bortezomib combination treatment in human cancer cells. U2OS-C3 osteosarcoma and MIA PaCa-2 pancreatic cancer cells were treated with DMSO or indicated concentrations of thiostrepton and borteozomib together for 24 hours. Cell lysates were immunoblotted for FoxM1, cleaved caspase-3 and β-actin as the loading control. (TIF) [file pone.0017110.s001.tif]
